# Supplementary material for: Printable microscale interfaces for long-term peripheral nerve mapping and precision control
Source: Nat Commun. 2020 Aug 21;11:4191. doi: 10.1038/s41467-020-18032-4 (PMC7442820; doi:10.1038/s41467-020-18032-4)
Supplement: Supplementary file 1 — Supplementary Information [file 41467_2020_18032_MOESM1_ESM.pdf]

Printable microscale interfaces for long-term peripheral nerve mapping and precision control

Otchy et al.

Supplementary Information

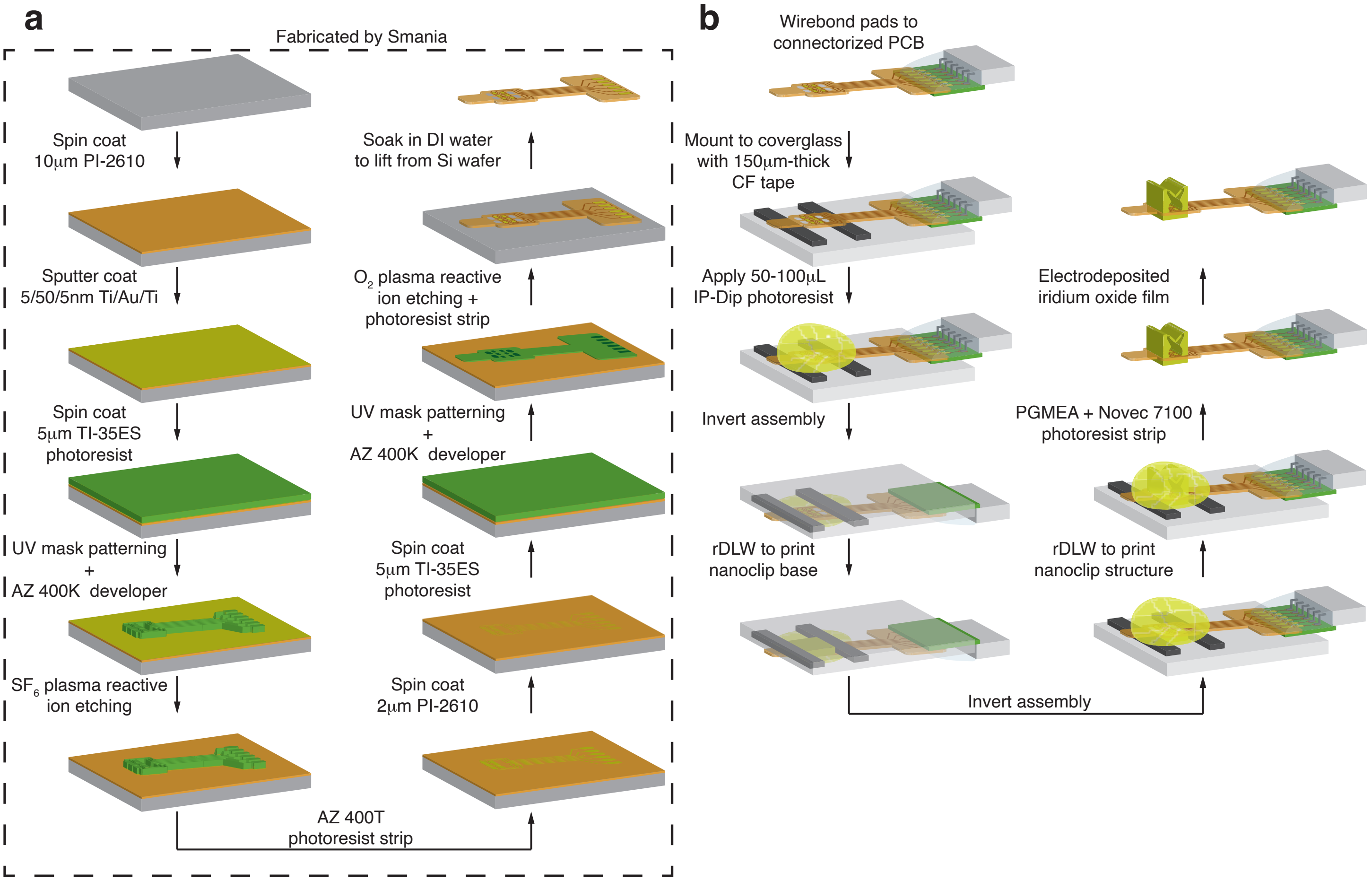

Supplementary Figure 1 I Nanoclip interface fabrication process. (a) Polyimide thin-film electrode fabrication process (produced by Smania). (b) Thin-film electrode and nanoclip integration process.

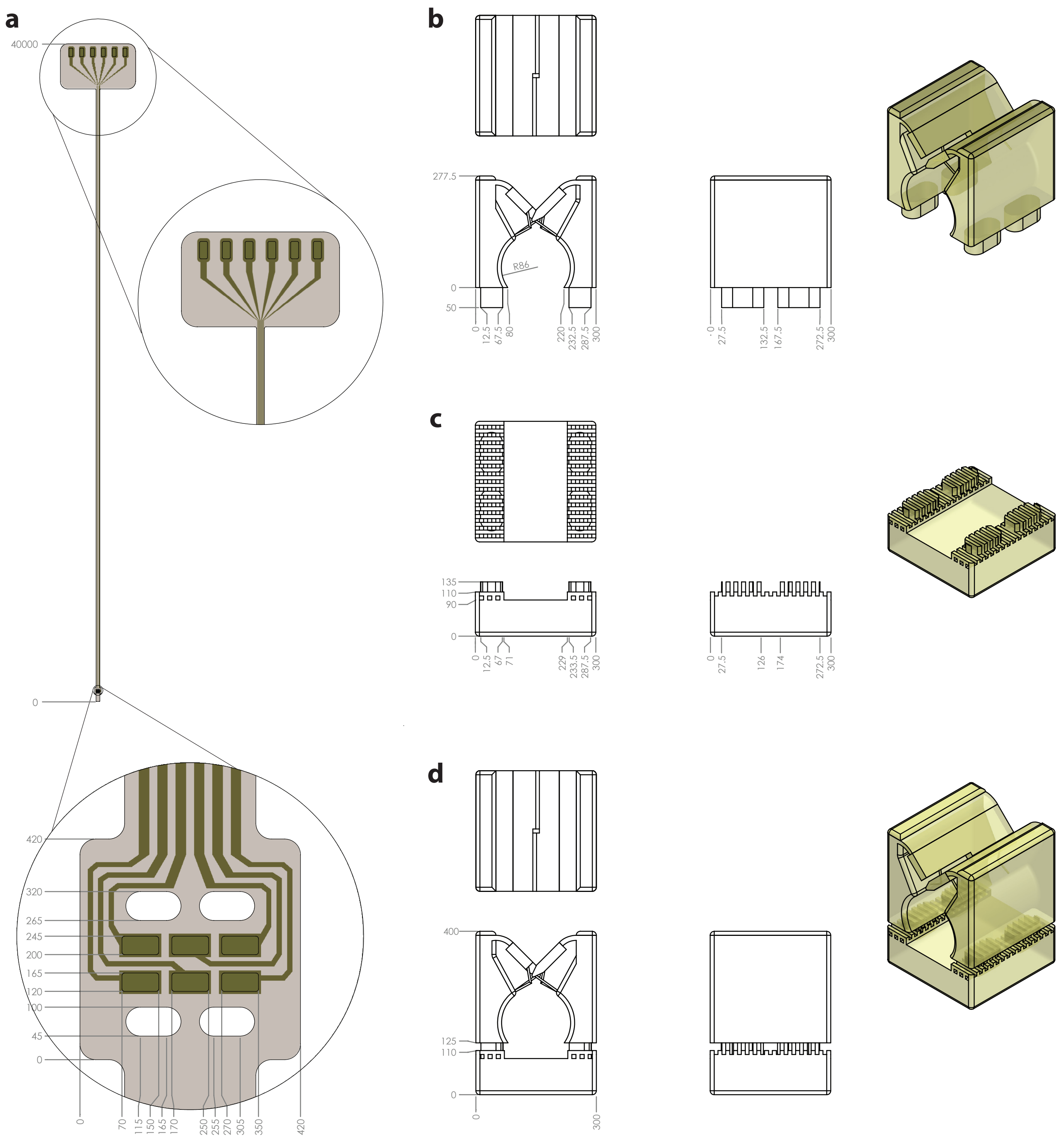

Supplementary Figure 2 l Design and geometry of the nanoclip interface components. (a) Diagram and dimensions of the polyimide thin-film electrode array. Top inset: Bottom inset: electrode array and through-vias. (b-d) Diagram and dimensions of the 3D-printed nanoclip: (b) upper structure comprising the interlocking trap doors and nerve cavity; (c) base structure with interleaved tenon; (d) as-fabricated integration of the nanoclip upper and base. All dimensions in microns.

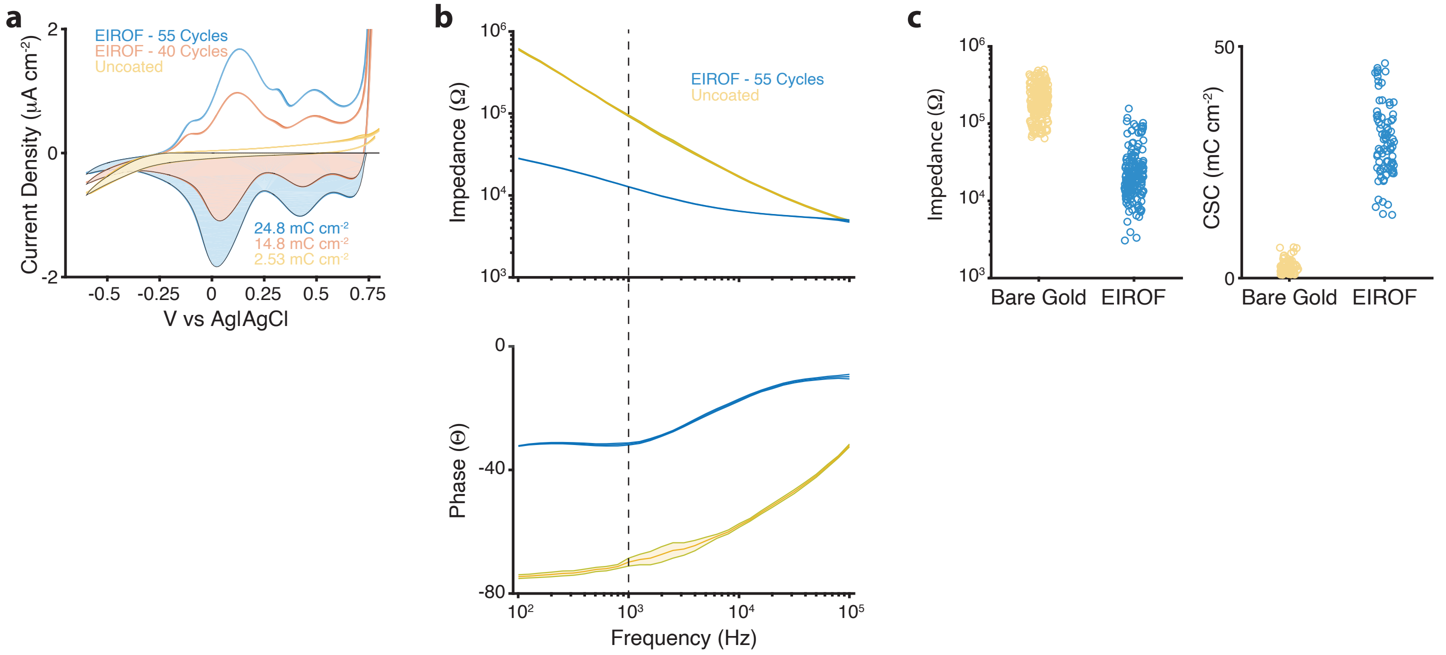

Supplementary Figure 3 | Electrodeposition of iridium oxide films to condition electrodes for neural recording and stimulation. (a) Representative cyclic voltammetry curves measured in PBS for a gold electrode before and after EIROF deposition. Shaded region shows time-integral of the negative current used to calculate charge storage capacity (CSC) as in (c) (Cogan, 2008). Yellow: uncoated gold. Red: 40 deposition cycles. Blue: 55 cycles. (b) Representative electrode impedance spectroscopy measured in PBS for a gold electrode before and after EIROF deposition. Top: Impedance versus frequency. Bottom: Phase versus frequency. Dashed line indicated nominal 1 kHz as in (c). Line color as in (a). Bold line shows mean; shaded region shows std across n = 3 repeated measurements. (c) Summary plots for impedance at 1 kHz (left) and CSC (right) before (yellow) and after (blue) IROF deposition (n = 210 pads in 35 devices and n = 90 pads in 15 devices, respectively). Data points correspond to individual electrode pads.

a

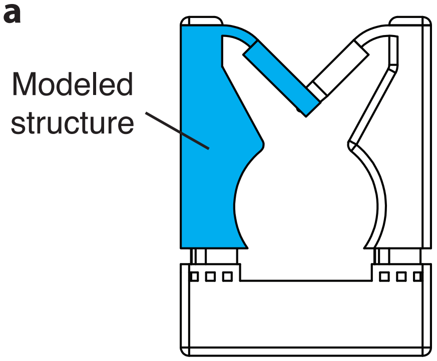

b

Material	Density (g cm^{-3})	Young's modulus (E) (GPa)	Yield strength (MPa)	Tensile strength (MPa)	Poisson's ratio
Parylene	1.3	2.8 - 5.2	55	69	0.4
Polyimide	1.3	2.5 - 4.1	230	345	0.37
PDMS	0.97	4.4 - 8.7 (x10^{-4})	2.3	5	0.5
IP-Dip	1.2	0.75 - 4.5	67	220	0.35

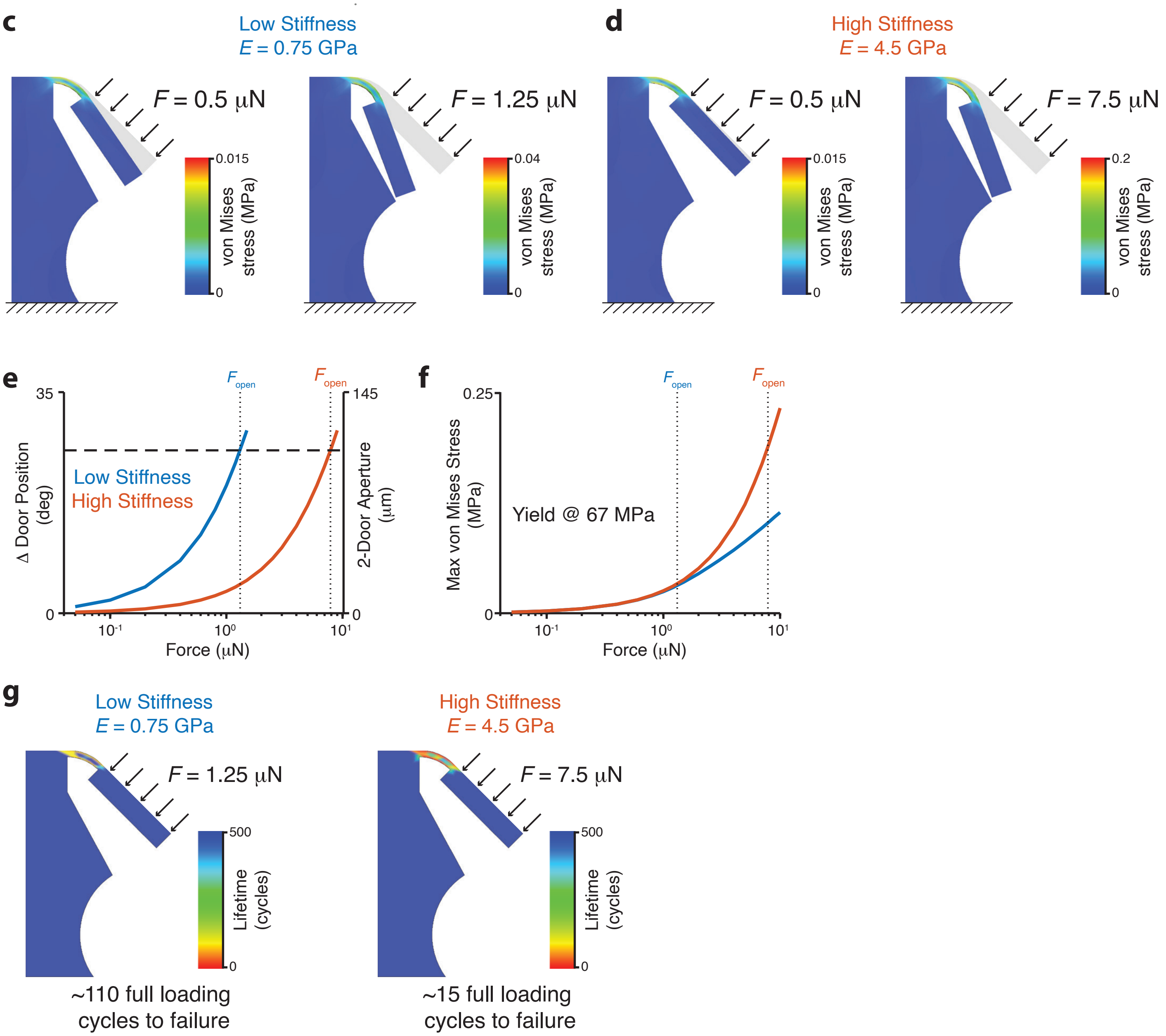

Supplementary Figure 4 | Finite element modeling of trap door actuation forces and fatigue. (a) Schematic of the printed nanoclip. The portion of the printed structure modeled in this analysis is shown in blue. (b) Table of mechanical properties of materials commonly used in neural interface fabrication. Yellow highlights the material used in the printed structure and the property values used in deformation and fatigue analyses. (c) Results of finite element modeling of trap door deformation under constant partial (left) and full loading forces (right) using the lower bound estimate for IP-Dip stiffness. Grey silhouette shows the initial, undeformed door and hinge position; overlying graded-color shows modeling estimates of both final position under prescribed loading and magnitude and distribution of von Mises stress within the structure. Black arrows represent actuation force applied normal to the door and evenly distributed across the surface; hatched base indicates the fixed surface in the model. The colorbar shows scaling for the von Mises stress heatmap. (d) Same as (c), but using higher bound estimate of IP-Dip stiffness. (e) Estimates of the change in trap door position as a function of actuation forces for both lower (blue) and higher (red) IP-Dip stiffness. Left axis shows the deflection of the door measured as the change in angle from initial position; right axis shows the width of opening created by deforming both trap doors equally. Dashed line denotes the maximum open position due to mechanical interference with the interior of the structure. Dotted lines indicate forces necessary to fully open doors in the low and high stiffness estimates. (f) Summary estimates of the maximum von Mises stress as a function of actuation forces for both lower (blue) and higher (red) IP-Dip stiffness estimates. Dotted lines are as in (e). Note that yield stress for IP-Dip (~67 MPa) is 2-3 orders greater than these estimates, suggesting low likelihood of failure under comparable loads. (g) Mechanical modeling estimates of trap door cycles to fatigue under full loading forces for both lower (left) and higher (right) stiffness estimates. The graded-color estimates number and location of cycles to failure in the printed structure.

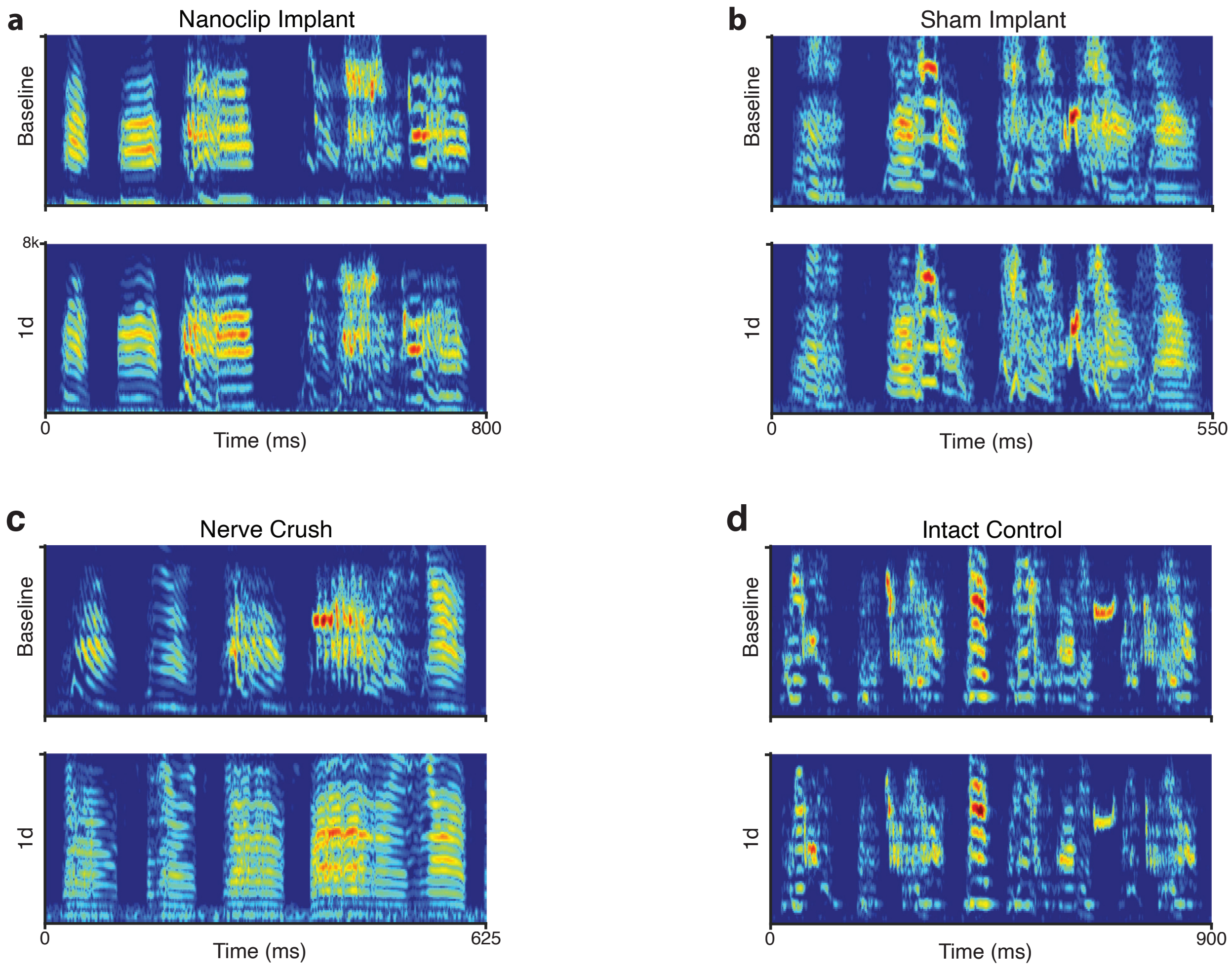

Supplementary Figure 5 l Representative song motifs recorded before and after nerve implant or manipulation. (a) Spectrogram of song motif recorded one day before (top) and one day after (bottom) bilateral nanoclip implant on nXIIts. (b) Same as (a) for bilateral sham implant. (c) Same as (a) for bilateral nerve crush. (d) Same as (a) for an intact/unmanipulated animal.

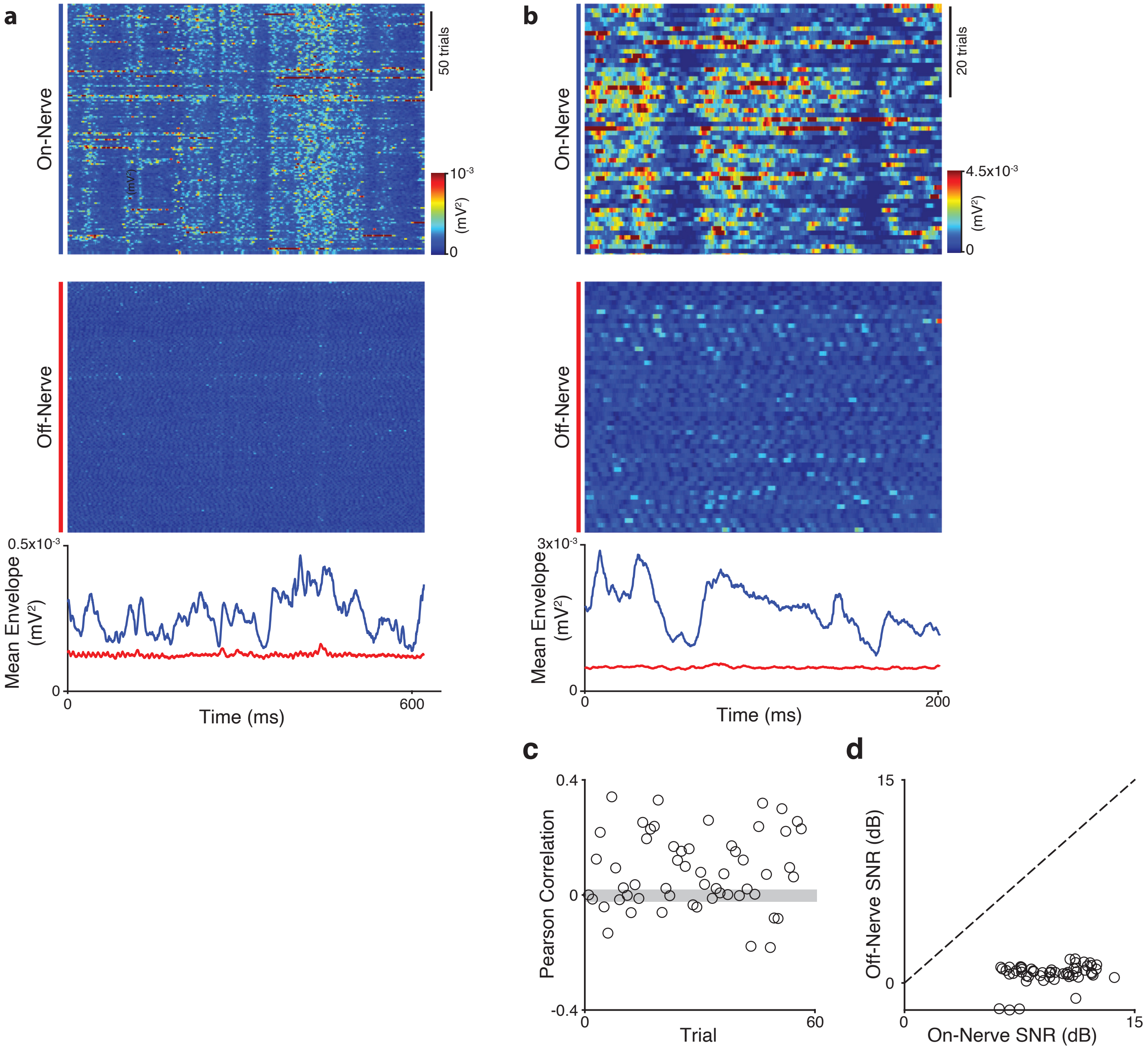

Supplementary Figure 6 | Additional data supporting the neuronal origin of recorded signals. (a) Simultaneous on-nerve and off-nerve recording envelopes aligned to song for the representative bird shown in Figure 5. (Top) Song-aligned on-nerve recording envelopes for motifs produced on a single day. (Middle) Song-aligned off-nerve recording envelopes for motifs recorded simultaneous to the those shown at top. Ordering and color-gradient are same as at top. (Bottom) Mean song-aligned envelopes for the on-nerve (blue) and off-nerve (red) recordings shown top and middle. (b) Same as in (a) for a second animal. (c) Trial-by-trial Pearson correlations between the on-nerve and off-nerve recordings for the bird shown in (b). Each circle represents an individual trial; the grey-shaded box indicates the 95% confidence interval for zero-correlation generated by bootstrap. (d) Trial-by-trial comparison of SNR for on-nerve and off-nerve recordings for the bird shown in (b). Each circle represents an individual trial; dashed line indicates unity.

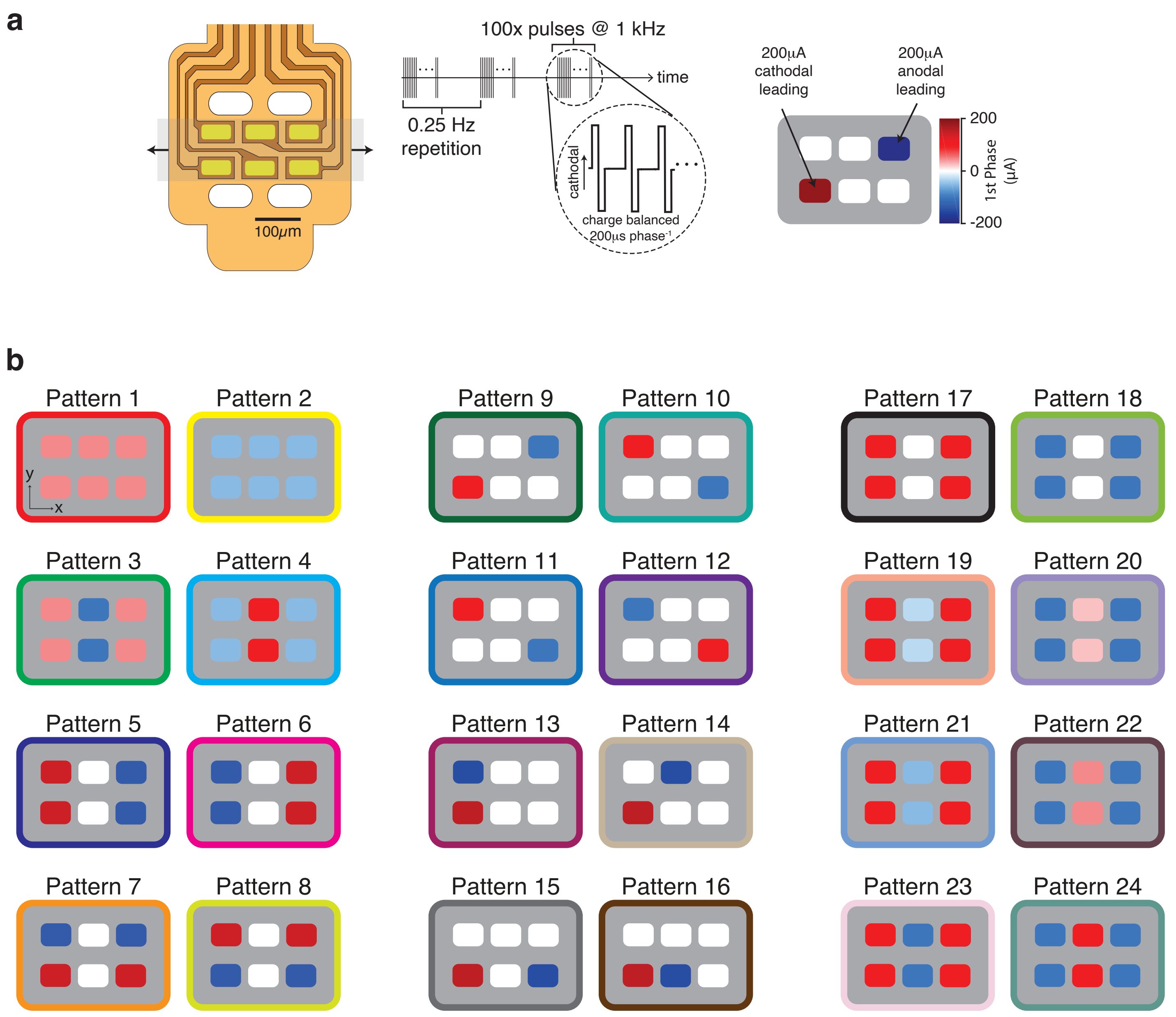

Supplementary Figure 7 I Current steering patterns for fictive singing experiments. (a) Schema for multi-channel stimulation patterns for fictive-singing experiments. (Left) Diagram of the six-channel electrode array used in all experiments. The shaded gray region shows the location of a retained nerve; the arrows indicate the nerve axis. (Right) 100 biphasic stimulating pulses, 200 µs phase^{-1} at -200-200 µA, were delivered at 1 kHz, repeated at 0.25 Hz. In the pattern schema, the color of each pad indicates the magnitude and direction of the leading phase of the stimulation pulse. (b) All current-steering stimulation patterns. The electrode pad color is as in (a). The color of the border indicates the stimulation patterns corresponding to data points in Figure 7c.
