# Supplementary material for: Printable microscale interfaces for long-term peripheral nerve mapping and precision control
Source: Nat Commun. 2020 Aug 21;11:4191. doi: 10.1038/s41467-020-18032-4 (PMC7442820; doi:10.1038/s41467-020-18032-4)
Supplement: Supplementary file 3 — Reporting Summary [file 41467_2020_18032_MOESM3_ESM.pdf]

nature research

Corresponding author(s): Timothy Otchy

Last updated by author(s): Jul 16, 2020

Reporting Summary

Nature Research wishes to improve the reproducibility of the work that we publish. This form provides structure for consistency and transparency in reporting. For further information on Nature Research policies, see our Editorial Policies and the Editorial Policy Checklist.

Statistics

For all statistical analyses, confirm that the following items are present in the figure legend, table legend, main text, or Methods section.

n/a	Confirmed	
☐	☒	The exact sample size (*n*) for each experimental group/condition, given as a discrete number and unit of measurement
☐	☒	A statement on whether measurements were taken from distinct samples or whether the same sample was measured repeatedly
☐	☒	The statistical test(s) used AND whether they are one- or two-sided<br>*Only common tests should be described solely by name; describe more complex techniques in the Methods section.*
☐	☒	A description of all covariates tested
☐	☒	A description of any assumptions or corrections, such as tests of normality and adjustment for multiple comparisons
☐	☒	A full description of the statistical parameters including central tendency (e.g. means) or other basic estimates (e.g. regression coefficient) AND variation (e.g. standard deviation) or associated estimates of uncertainty (e.g. confidence intervals)
☐	☒	For null hypothesis testing, the test statistic (e.g. *F*, *t*, *r*) with confidence intervals, effect sizes, degrees of freedom and *P* value noted<br>*Give P values as exact values whenever suitable.*
☒	☐	For Bayesian analysis, information on the choice of priors and Markov chain Monte Carlo settings
☒	☐	For hierarchical and complex designs, identification of the appropriate level for tests and full reporting of outcomes
☐	☒	Estimates of effect sizes (e.g. Cohen's *d*, Pearson's *r*), indicating how they were calculated

Our web collection on statistics for biologists contains articles on many of the points above.

Software and code

Policy information about availability of computer code

Data collection	All custom MATLAB (2018b) and LABView (2017) code is accessible in an online repository at https://github.com/timotchy/Otchy-et-al-2020
Data analysis	All custom MATLAB (2018b) code is accessible in an online repository at https://github.com/timotchy/Otchy-et-al-2020

For manuscripts utilizing custom algorithms or software that are central to the research but not yet described in published literature, software must be made available to editors and reviewers. We strongly encourage code deposition in a community repository (e.g. GitHub). See the Nature Research guidelines for submitting code & software for further information.

Data

Policy information about availability of data

All manuscripts must include a data availability statement. This statement should provide the following information, where applicable:

- Accession codes, unique identifiers, or web links for publicly available datasets
- A list of figures that have associated raw data
- A description of any restrictions on data availability

The source data underlying Figures 2d, 3e, 4e, 5e, and 7d are provided as a Source Data File. Data supporting the findings of this study are available at https://github.com/timotchy/Otchy-et-al-2020. All other data is available from the corresponding author upon request.

Field-specific reporting

Please select the one below that is the best fit for your research. If you are not sure, read the appropriate sections before making your selection.

☒ Life sciences ☐ Behavioural & social sciences ☐ Ecological, evolutionary & environmental sciences

For a reference copy of the document with all sections, see nature.com/documents/nr-reporting-summary-flat.pdf

Life sciences study design

All studies must disclose on these points even when the disclosure is negative.

Sample size	For all behavioral and electrophysiology experiments, the number of birds was determined based on previous publications using sonbirds to validate new interfacing methods (Lissandrello et al 2018, Gillis et al 2019, Otchy and Olveczky 2012, Leonardo and Fee 2005, and Liberti et al 2016).
Data exclusions	No animals or experimental data were excluded from experiments post-hoc.
Replication	We consider each recorded or manipulated animal as an independent sample, and all figure except 5e reflect n >= 3 biologically independent replicates. (Figure 5e contain replication in 2 animals.) The experimental outcomes were consistent across recordings and animals. Experiments were replicated in 3 birds for acute recordings, in 5 birds for chronic recordings, in 2 birds for on-off nerve control recordings, and 6 birds for fictive singing. Each nerve manipulation condition was replicated in 3 birds.
Randomization	The investigators randomly allocated animals from the Boston University breeding colony to experiments. No additional randomization of animal subject was warranted.
Blinding	The investigators were not blinded to allocation of animals during experiments and outcome assessment. As our studies focus on the in vivo validation of an implantable neural interface, it was not possible to conceal the presence or absence of the data collection device under study from the investigators.

Reporting for specific materials, systems and methods

We require information from authors about some types of materials, experimental systems and methods used in many studies. Here, indicate whether each material, system or method listed is relevant to your study. If you are not sure if a list item applies to your research, read the appropriate section before selecting a response.

Materials & experimental systems

n/a	Involved in the study	
☒	☐	Antibodies
☒	☐	Eukaryotic cell lines
☒	☐	Palaeontology and archaeology
☐	☒	Animals and other organisms
☒	☐	Human research participants
☒	☐	Clinical data
☒	☐	Dual use research of concern

Methods

n/a	Involved in the study	
☒	☐	ChIP-seq
☒	☐	Flow cytometry
☒	☐	MRI-based neuroimaging

Animals and other organisms

Policy information about studies involving animals; ARRIVE guidelines recommended for reporting animal research

Laboratory animals	male zebra finches (Taeniopygia guttata); 90+ days after hatching (i.e., adults); n = 37 birds in total
Wild animals	The study does not include wild animals
Field-collected samples	The study does not include field samples
Ethics oversight	Boston University Institutional Animal Care and Use Committee; animal care guidelines from the National Institutes of Health

Note that full information on the approval of the study protocol must also be provided in the manuscript.
